# Gut Bacteriome Modulation in *Rhipicephalus microplus* Under Biotic Stress From *Metarhizium anisopliae* and *Anaplasma marginale*

**DOI:** 10.1111/1758-2229.70455

**Published:** 2026-11-10

**Authors:** Thaís Almeida Correa, Elianne Piloto‐Sardiñas, Lucas Amoroso Lopes de Carvalho, Patrícia Gonzaga Paulino, Daniel Guariz Pinheiro, Isabele da Costa Angelo, Vânia R. E. P. Bittencourt, Alejandro Cabezas‐Cruz, Huarrisson Azevedo Santos, Patrícia Silva Golo

**Affiliations:** ^1^ Postgraduate Program in Veterinary Sciences Veterinary Institute, Federal Rural University of Rio de Janeiro Seropédica Rio de Janeiro Brazil; ^2^ Direction of Animal Health National Center for Animal and Plant Health, Carretera de Tapaste y Autopista Nacional San José de las Lajas Mayabeque Cuba; ^3^ Postgraduate Program in Agricultural and Livestock Microbiology, School of Agricultural and Veterinary Sciences São Paulo State University (UNESP) Jaboticabal São Paulo Brazil; ^4^ Department of Epidemiology and Public Health Veterinary Institute, Federal Rural University of Rio de Janeiro Seropédica Rio de Janeiro Brazil; ^5^ Laboratory of Bioinformatics, Department of Agricultural Sciences, Livestock and Environmental Biotechnology, School of Agricultural and Veterinary Sciences São Paulo State University (UNESP) Jaboticabal São Paulo Brazil; ^6^ Department of Animal Parasitology Veterinary Institute, Federal Rural University of Rio de Janeiro Seropédica Rio de Janeiro Brazil; ^7^ ANSES, INRAE, Ecole Nationale Vétérinaire d’Alfort UMR BIPAR, Laboratoire de Santé Animale Maisons‐Alfort France

**Keywords:** biological control, biotic stressors, ecological interactions, entomopathogenic fungi, microbial ecology, network reorganization

## Abstract

Understanding how multiple biotic stressors shape host‐associated bacteriomes is essential for predicting ecological outcomes. This study examined interactions among the cattle tick *Rhipicephalus microplus* , the pathogen *Anaplasma marginale* and the entomopathogenic fungus *Metarhizium anisopliae*. Across treatments involving fungal exposure alone or combined with *A. marginale* , the gut bacteriome comprised 698 ASVs, of which 28.2% formed a stable core dominated by Bacteroidota and Bacillota. Fungal‐associated stress drove functional shifts, including enrichment of SCFA‐producing taxa such as *Akkermansia muciniphila* and *Lachnospiraceae*, indicating reorganization preceding major taxonomic changes. Co‐occurrence networks were restructured under fungal and combined pressures, showing high connectivity, predominance of negative correlations and antagonistic interactions driven by keystone taxa like *Prevotella*. The combined‐stressor network showed greater robustness, cohesion and communication efficiency than single‐stressor conditions. Functional predictions revealed conserved metabolic profiles, yet co‐exposure increased functional diversity and gene abundance, with *A. marginale* exerting dominant influence. In silico removal of *A. marginale* from the co‐exposure condition indicated a persistent ecological imprint, preventing reversion to the basal fungal‐only configuration and retaining structural traces of prior colonization. Overall, simultaneous pathogen–fungus pressures reorganize microbial networks, tightening interactions and enhancing stability, underscoring the dual role of *M. anisopliae* as a biocontrol agent and potential modulator of bacteriome resilience.

## Graphical abstract

(A) Ticks exposed to *Metarhizium anisopliae* and *Anaplasma marginale* showed enriched SCFA‐producing bacteria, restructured microbial networks and greater robustness under combined stress. Co‐exposure increased functional profiles and KEGG orthologl abundance, with *A. marginale* exerting a dominant influence. (C) In silico removal of the pathogen revealed a persistent ecological imprint on network structure, preventing network reversion and suggestingg that simultaneous biotic stressors affect bacteriome stability.

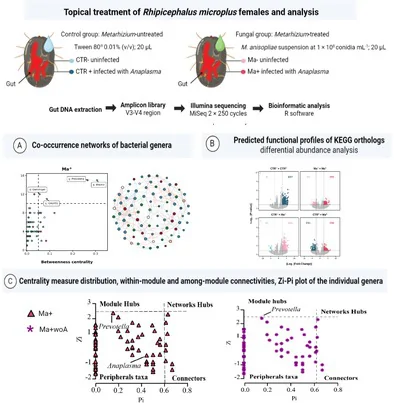

## 1 Introduction

The cattle tick, *Rhipicephalus microplus* (Canestrini 1887), acts both as a debilitating hematophagous ectoparasite and a vector of hemoparasites (de la Fuente et al. 2008; Barré and Uilenberg 2010). The combined effects of parasitism and pathogen transmission by *R. microplus* lead to substantial economic losses. In Brazil, the last comprehensive estimate reported annual losses of US$3.24 billion (Grisi et al. 2014).

Among the pathogens vectored by *R. microplus* , *Anaplasma marginale* (Rickettsiales: Anaplasmataceae) (Dumler et al. 2001) is a gram‐negative obligate intracellular bacterium, associated with bovine anaplasmosis and is one of the agents responsible for cattle tick fever (Aubry and Geale 2011; Puentes and Riet‐Correa 2023). The tick midgut represents the primary site of pathogen entry and colonization and harbours microbial communities composed of symbionts, commensals and pathogens (Berg et al. 2020; Bonnet and Pollet 2021; Hernandez et al. 2023). Previous studies have demonstrated that changes in the tick midgut microbiota may influence vector competence and pathogen persistence. The composition of the tick midgut microbiota is not static and can be shaped by environmental conditions (Gurfield et al. 2017; Brinkerhoff et al. 2020) and host‐related factors, including antibiotic exposure during blood feeding (Zhong et al. 2007; Narasimhan et al. 2014; Guizzo et al. 2022; Mesquita et al. 2023). These studies demonstrate that external perturbations can significantly alter microbial organization in ticks, reinforcing the role of the microbiota as a dynamic interface between vector and pathogen.

Despite increasing evidence that pathogens can influence tick‐associated microbial communities and that entomopathogenic fungi can alter tick physiology and immune responses, these factors have been investigated separately. To date, no study has evaluated the tripartite interaction among *R. microplus* , *A. marginale* and *Metarhizium anisopliae* at the gut bacteriome level. Understanding how fungal exposure influences the microbial organization of ticks already infected with *A. marginale* may provide important ecological insights into vector competence, microbial network organization and the unintended effects of biological control agents on pathogen‐associated bacteriomes.

One such agent is the entomopathogenic fungus *M. anisopliae*, increasingly explored as a biocontrol alternative to chemical acaricides. Entomopathogenic fungi have emerged as promising alternatives for tick control, with several studies demonstrating the effectiveness of *M. anisopliae* against *R. microplus* (Camargo et al. 2016; Mesquita et al. 2020; Marciano et al. 2021; Meirelles et al. 2023). Nevertheless, it remains unknown whether fungal infection alters the gut bacteriota of ticks already infected with *A. marginale* . Addressing this gap may improve our understanding of how biological control agents interact with pathogen‐associated bacterial communities in arthropod vectors.

Advanced sequencing techniques such as next‐generation sequencing (NGS) enable the examination of vast quantities of DNA fragments using a high‐capacity sequencing approach (Morozova and Marra 2008; Greay et al. 2018). Recently, other authors have confirmed the variability of the *R. microplus* midgut microbiota according to the region: Colombia (Segura et al. 2020), Brazil (Guizzo et al. 2022) and China (Zhang et al. 2022). Piloto‐Sardiñas et al. (2024) explored the temporal changes in the microbiome of *R. microplus* over 2 years, focusing on its interaction with *A. marginale* . These findings emphasize the significant influence of *A. marginale* on the tick microbial community. These insights highlight the complexity of host‐pathogen‐bacteriota interactions and reinforce the importance of studying how external agents may disrupt or reshape this balance.

The present study aimed, for the first time, to analyse the combined influence of *A. marginale* infection and *M. anisopliae* exposure on the gut bacteriome of *R. microplus* . We evaluated changes in microbial diversity, community organization, co‐occurrence network patterns and inferred functional profiles to better understand how pathogen infection and fungal exposure may interact within the tick microbiome.

## 2 Methods

### 2.1 Calf Selection and Tick Collection

One calf naturally infected with *A. marginale* , confirmed by Nested PCR (nPCR) according to de Echaide et al. (1998), was selected for artificial infestation with *R. microplus* larvae. The *A. marginale* ‐positive calf was artificially infested controlled with *R. microplus* larvae originating from a colony maintained at the Laboratory of Microbial Control of Arthropods of Veterinary Importance at the Federal Rural University of Rio de Janeiro (UFRRJ) under controlled conditions and routinely screened to ensure the absence of common bovine hemoparasites. After 21 days of feeding, engorged female ticks were manually collected directly from the animal's body. All procedures were approved by the Ethics Committee on the Use of Animals of the UFRRJ/Veterinary Institute (protocols no. 4832020623 and 6274250624). Additional methodological details are provided in Supporting Information (Section S1.1).

### 2.2 *Metarhizium anisopliae* Fungal Suspension

*Metarhizium anisopliae* LCM S04 (Mesquita et al. 2020) was used in this study. This soil‐borne Brazilian native isolate (Correa et al. 2022) was maintained in the Collection of Entomopathogenic Fungal Cultures of the Laboratory of Microbial Control of Arthropods (CCFELCM), UFRRJ. As the present study assessed Brazilian genetic heritage, the research was registered with the National System for the Management of Genetic Heritage and Associated Traditional Knowledge (Sisgen) under the code AA47CB6. Conidia were scraped after 14 days of culture medium and suspended in sterile distilled water solution containing 0.01% (v/v) polyoxyethylene sorbitan monooleate‐Tween 80 (Sigma‐Aldrich, St. Louis, MO, USA). The fungal concentration was adjusted to 1 × 10^8^ conidia. mL^−1^ and conidial viability was evaluated according Lopes et al. (2013). Additional methodological details are provided in Supporting Information (Section S1.2).

### 2.3 Fungal Treatment in *R. microplus* Females

Tick females were initially divided into two groups with 15 ticks each: CTR (control) (ticks topically treated with Tween 80 0.01% (v/v); 20 μL) and Ma (ticks topically treated with 20 μL of *M. anisopliae* suspension at 1 × 10^8^ conidia mL^−1^). The treatment was applied to the dorsal region of the tick and the ticks were kept at 25°C ± 1°C and RH ≥ 80%. Seventy‐two hours after treatment, the guts of individual females from each group were dissected for DNA extraction.

### 2.4 *R. microplus* Dissection and Gut DNA Extraction

The guts of *R. microplus* females were dissected using sterile tweezers and scalpel blades using iced sterile NaCl 0.9%. The removed gut tissues were washed twice in sterile saline solution and stored in RNA later (Thermo Fisher Scientific, Waltham, MA, USA) at −80°C until extraction. Each tick was dissected according to a protocol adapted from Tidwell et al. (2021). First, the guts were washed with 1 mL phosphate‐buffered saline (PBS) and centrifuged for 5 min. This process was repeated three times to completely remove the RNA later reagent. The tick guts were frozen in liquid nitrogen and macerated using sterile pestles. The DNA of the gut homogenate was extracted using the DNeasy Blood & Tissue kit (QIAGEN, São Paulo, Brazil), according to the manufacturer's instructions.

### 2.5 Analysis of the DNA Quality and Presence of *A. marginale* in the Experimental Groups

DNA concentration and purity from tick gut samples were evaluated using a NanoDrop spectrophotometer (Thermo Fisher Scientific, Waltham, MA, USA) and DNA integrity was assessed by electrophoresis on a 1% agarose gel. The *Anaplasma marginale* DNA was detected using qPCR targeting the *msp* 1β gene according to Carelli et al. (2007). Relative pathogen load was normalized using the *R. microplus* 18S rRNA endogenous gene described by Saldivar et al. (2008). All reactions were performed in triplicate using the StepOnePlus Real‐Time PCR System (Applied Biosystems, Thermo Fisher Scientific, Waltham, MA, USA). Additional information regarding primers, reaction mixtures and thermocycling conditions is provided in Supporting Information (Section S1.3; Table S1).

### 2.6 Detection of *M. anisopliae* in the Gut of *R. microplus* Female

The presence of *M. anisopliae* DNA in tick gut samples was evaluated by qPCR using the same DNA samples described in Section 2.5. Amplification targeted the *EF‐1α* gene according to Nijhof et al. (2009). Reactions were performed in triplicate using the StepOnePlus Real‐Time PCR System (Applied Biosystems, Thermo Fisher Scientific, Waltham, MA, USA). DNA extracted from *M. anisopliae* cultured on oat medium was used as the positive control, while molecular‐grade water was used as the negative control. Additional methodological details, including primers, reaction conditions and melt curve analysis parameters, are described in Supporting Information (Section S1.4; Table S1).

### 2.7 Study Design

The *R. microplus* gut bacteriome was characterized by 16S rRNA gene sequencing. Experimental groups were established under four conditions: guts from *Metarhizium*‐untreated females, either uninfected (CTR−) or infected with *Anaplasma* (CTR+); and guts from *Metarhizium*‐treated females, either uninfected (Ma−) or infected with *Anaplasma* (Ma+). Tick guts testing positive for *Anaplasma* were assigned to the infected groups (+). All ticks exposed to *Metarhizium*, irrespective of fungal detection within the gut, were assigned to the treated groups. DNA extracted from individual tick gut samples was used to construct 16S rRNA V3–V4 region amplicon libraries (Abraham et al. 2017) and sequencing was carried out on the Illumina MiSeq platform (MiSeq Reagent Kit V2 Nano, 2 × 250 cycles).

### 2.8 Processing of Raw Reads

Raw reads were quality filtered, trimmed, merged and processed using a DADA2‐based pipeline implemented in R according to Callahan et al. (2016). Amplicon sequence variants (ASVs) were inferred, taxonomically classified using the Greengenes2 database described by McDonald et al. (2024) and screened for potential contaminants using the Decontam package according to Davis et al. (2018). Phylogenetic reconstruction and secondary taxonomic verification were additionally performed to support ASV classification. Detailed bioinformatic parameters, filtering criteria and sequence processing steps are provided in Supporting Information (Section S1.5).

### 2.9 Microbiome Analysis

Alpha diversity was assessed using richness, Pielou's evenness and Faith's phylogenetic diversity indices, whereas beta diversity was evaluated using weighted UniFrac distances. Differentially abundant taxa were identified using LEfSe for biomarker discovery (Segata et al. 2011).

Microbial co‐occurrence networks were inferred using SparCC correlations implemented in SpiecEasi (Kurtz et al. 2015) and network topology analyses were conducted using igraph (Csardi and Nepusz 2006). Local connectivity patterns, node centrality distributions and network robustness under node removal and node addition perturbations were also evaluated to characterize microbial community organization.

The functional potential of bacterial communities was inferred using PICRUSt2 (Douglas et al. 2019) and differential functional abundance was analysed using DESeq2 (Love et al. 2014). Detailed analytical parameters, filtering thresholds and statistical settings are provided in Supporting Information (Section S1.6).

### 2.10 Statistical Analyses

For general statistical comparisons, two‐way ANOVA was used, considering the factors of treatment and *A. marginale* infection status based on ranked values (‘ANOVA on ranks’), as proposed by Sawilowsky (1990). To evaluate the significant differences between the treatment means, a confidence level of 95% (*p* < 0.05) was adopted. For variables that presented significant differences, multiple comparisons were performed using Fisher's LSD post hoc test using the R package, agricolae (de Mendiburu 2023). Differences in beta diversity between groups were evaluated using Permutational Multivariate Analysis of Variance (PERMANOVA) with the ‘adonis2’ function of the R package vegan. Post hoc pairwise comparisons were conducted using the ‘pairwise.adonis’ function of the R package (Arbizu 2017). Multidimensional distances were visualized using Principal Coordinate Analysis (PCoA). Graphical representations were created using the R package ggplot2 (Wickham 2016). Heatmaps of taxonomic and functional data were generated using the R package ComplexHeatmap (Gu et al. 2016).

## 3 Results

### 3.1 Molecular Confirmation of *A. marginale* and *M. anisopliae* Infection in Early Fungal‐Bacterial Interaction Scenarios

qPCR was performed to validate the infection status of the tick samples and detect *A. marginale* and *M. anisopliae*. The infection profiles obtained correspond to the first 72 h. The fungal suspensions used in the experiments had a conidia viability of at least 95%, ensuring the effectiveness of the treatment. Among the samples in the infected control group (CTR+), nine out of 15 gut samples were positive for *A. marginale* . In the group treated with *M. anisopliae* and infected with *A. marginale* (Ma+), six out of 15 samples were positive for *A. marginale* . In the groups treated with fungi, only two gut samples from Ma+ tested positive for *M. anisopliae*.

These molecular confirmations demonstrate that the analytical approach was sufficient to detect both fungal exposure and *A. marginale* infection. During the study, all ticks exposed to *M. anisopliae*, whether positive or not to the fungus, were considered treated (Ma). From an ecological perspective, the term ‘treated’ will be used hereafter as a synonym for exposure, regardless of whether intestinal colonization was detected. Exposure alone can influence the bacteriome and initiate nascent ecological interactions.

### 3.2 The Gut Bacteriome of *R. microplus* Maintains Bacterial Community Diversity and Structure Under *A. marginale* Infection and Early‐Stage From *M. anisopliae* Exposure

Gut bacterial composition of all ticks' samples revealed a total of 698 ASVs, of which 197 (28.22%) were common to all groups. PCoA analysis indicated no significant differences in the composition of gut microbial communities among the analysed groups (PERMANOVA, *p* > 0.05). Alpha diversity was assessed using three metrics (Richness, Pielou's evenness and Faith's phylogenetic diversity) and no significant differences in alpha diversity were observed between the treatments (*p* > 0.05) (Supporting Information, Section S2.1; Figures S1 and S2).

### 3.3 Shared Taxa Predominate in the Gut Bacteriome of *R. microplus* Across Experimental Groups Treated With *M. anisopliae* and Infected With *A. marginale*

Taxonomic assignment was highly efficient, with 98.8% of sequences classified at the genus level. Overall, gut bacterial communities were largely dominated by shared taxa across all experimental groups, with no phylum‐level exclusivity observed and a high proportion of genera shared among treatments (Supporting Information, Section S2.2; Figures S3 and S4).

LefSe analysis (Figure 1) identified significant biomarkers, representing 3.15% of the total ASVs analysed (698 ASVs). Interesting, in groups exposed to the fungus (Ma− and Ma+), several biomarkers corresponded to well‐known short‐chain fatty acid (SCFA) producers, including *Faecalibacterium prausnitzii* and *Bacteroides stercoris* in Ma− and *Akkermansia muciniphila* and members of *Lachnospiraceae* in Ma+.

**FIGURE 1:**
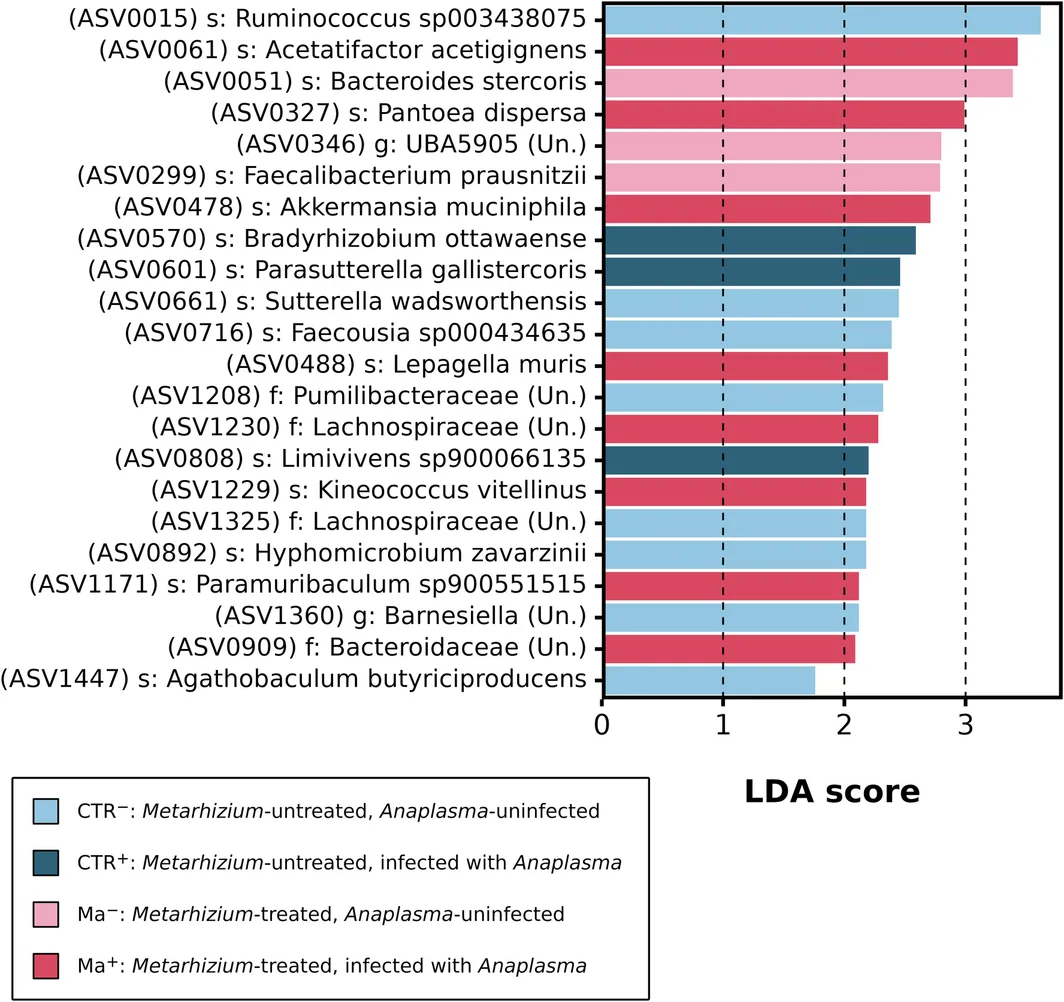
Taxonomic biomarker identified by LEfSe analysis at the ASV level. The bar graph shows the taxonomic biomarkers identified by Linear Discriminant Analysis Effect Size (LEfSe) analysis, with the best taxonomic classifications obtained for the ASVs considered biomarkers. The colours of the bars indicate the treatment group that is most strongly associated with each biomarker. The displacement along the X‐axis represents the Linear Discriminant Analysis (LDA) values. Statistical thresholds: *p* < 0.05 and LDA > 1.5.

### 3.4 Bacterial Assembly Patterns in the Gut of *R. microplus* Vary Under *A. marginale* Infection and Early‐Stage From *M. anisopliae* Exposure

The CTR− network represents the assembly of the bacterial community associated with the gut of *R. microplus* without selection pressure involving *M. anisopliae* exposure and infection by *A. marginale* . The network presented the highest number of nodes (92) and edges, with a predominance of negative correlations (57.23%). This structure was reflected in its centrality metrics, where it exhibited the second highest average Degree (3.457 ± 2.201) and a high average Betweenness (0.032 ± 0.046) (Table 1). Keystone analysis for this group identified *Phocaeicola* and *Faecalibacterium* as key genera (Figure 2). Overall, the network pattern is consistent with the absence of both selection pressures. Furthermore, the predominance of negative interactions suggests a state of basal negative interaction in the community in the absence of external selective forces.

**TABLE 1:** Topological characteristics and centrality measures of the co‐occurrence networks of the genera present in the treatments.

Parameters	CTR−	CTR+	Ma−	Ma+
Number of Nodes	92	72	53	85
Number of Edges	159	69	49	193
Positives Edges	68 (42.77%)	41 (59.42%)	26 (53.06%)	86 (44.56%)
Negative Edges	91 (57.23%)	28 (40.58%)	23 (46.94%)	107 (55.44%)
Number of Clusters	3	9	12	3
Modularity Coefficient	0.0372	0.457	0.8013	0.0702
Diameter	9	12	6	10
Average Degree	3.457 (±2.201)^b^	1.917 (±1.361)^c^	1.849 (±1.045)^c^	4.541 (±3.115)^a^
Average Betweenness	0.032 (±0.046)^a^	0.027 (±0.057)^b^	0.003 (±0.005)^c^	0.027 (±0.048)^a^
Average Closeness	0.276 (±0.152)^c^	0.387 (±0.302)^c^	0.606 (±0.248)^a^	0.329 (±0.154)^b^

*Note:* Comparison of average values of Degree, Betweenness and Closeness of bacterial co‐occurrence networks between treatment groups. Statistical analysis was performed using two‐way ANOVA on ranks (*p* < 0.05), with group differences indicated by different letters based on Fisher's LSD post hoc test. Treatments: (CTR−) *Metarhizium*‐untreated, uninfected with *Anaplasma*; (CTR+) *Metarhizium*‐untreated, infected with *Anaplasma*; (Ma−) *Metarhizium*‐treated (1 × 10^8^ conidia. mL^−1^), uninfected with *Anaplasma*. (Ma+) *Metarhizium*‐treated (1 × 10^8^ conidia. mL^−1^), infected with *Anaplasma*.

**FIGURE 2:**
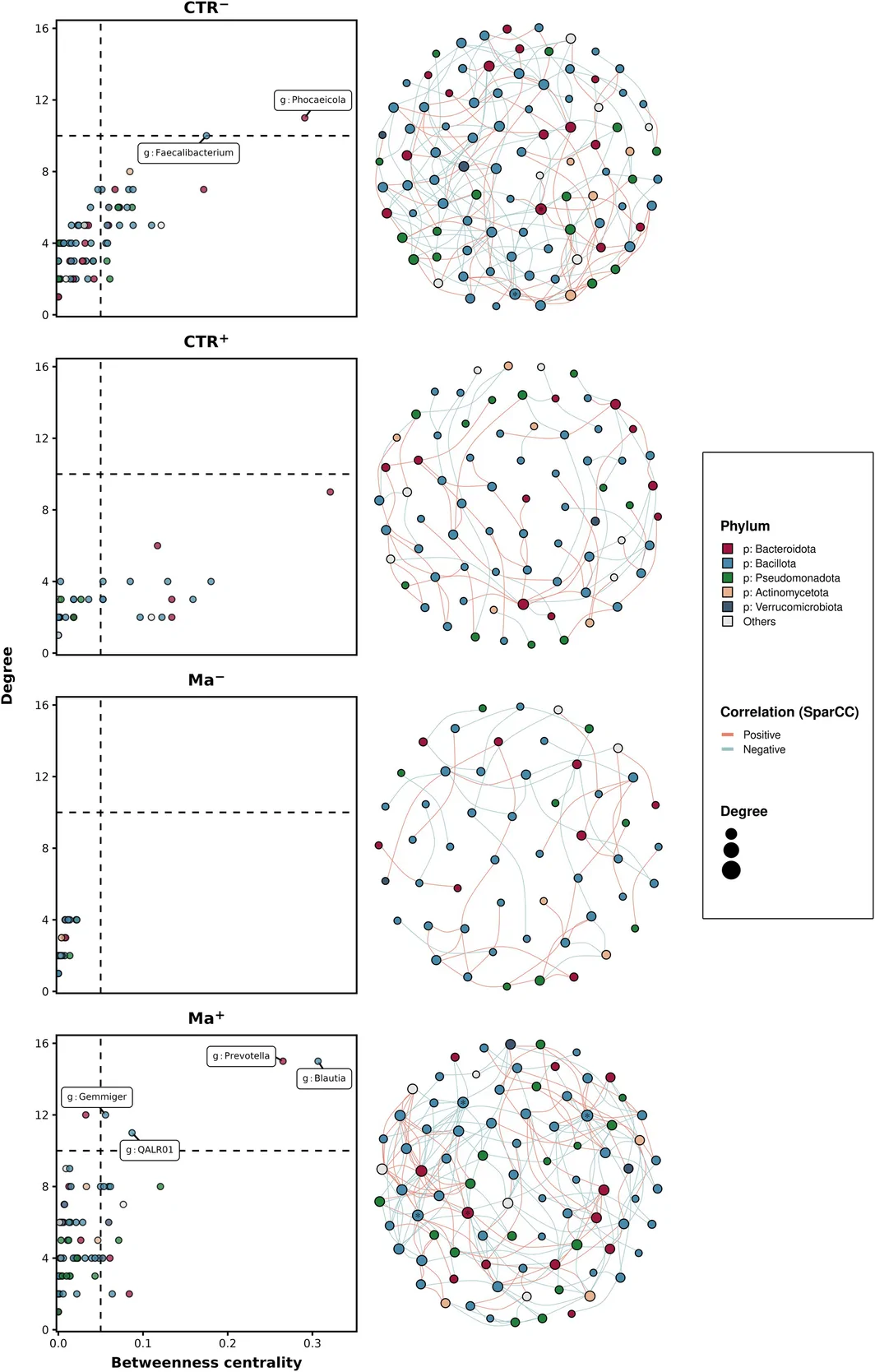
Impact of early ecological interactions, resulting from exposure to *Metarhizium anisopliae* and infection by *Anaplasma marginale* , on the assembly of the bacterial community in the gut of *Rhipicephalus microplus* . Co‐occurrence networks of bacterial genera between the treatments. Co‐occurrence networks representing positive (red) and negative (blue) correlations between bacterial genera in each treatment group. Correlations were determined using Sparse Correlations for Compositional Data (SparCC) analysis with statistical thresholds of *p* < 0.05, SparCC > 0.5 for positive correlations or < −0.05 for negative correlations. Keystone analysis with degrees of linkages (degree) > 10 and betweenness centrality (betweenness centrality) > 0.05 are marked with asterisks in the network (right) and identified in the dot plot correlating these two parameters (left). Treatments: (CTR−) *Metarhizium*‐untreated, uninfected with *Anaplasma*; (CTR+) *Metarhizium*‐untreated, infected with *Anaplasma*; (Ma−) *Metarhizium*‐treated, uninfected with *Anaplasma*. (Ma+) *Metarhizium*‐treated, infected with *Anaplasma*.

With the presence of *A. marginale* (CTR+), although it presented 72 nodes, this network was distinguished by an inversion in interactions, showing a higher proportion of positive edges (59.42%), an average Degree (1.917 ± 1.361) and Betweenness (0.027 ± 0.057) statistically different from CTR− (Table 1). Interestingly, the CTR+ network presented a higher number of clusters and a significantly higher modularity coefficient compared to CTR−, indicating greater network fragmentation.

Regarding treatments involving *Metarhizium*, the Ma− group stood out for its most fragmented network structure, with 53 nodes, but organized into 12 clusters and presenting the highest modularity coefficient (0.801) and smallest diameter (6). Centrality metrics confirmed this characteristic, with Ma− recording the highest average Closeness value (0.606 ± 0.248), indicating that nodes were, on average, closer to each other (Table 1).

In contrast to Ma−, the Ma+ network (double selective pressure: *Metarhizium* exposure and *A. marginale* infection) exhibited a larger and more connected structure, with the highest average degree of connections, yet dominated by negative correlations (Table 1). Genera such as *Prevotella, Blautia, Gemmiger* and QALR01 were identified as keystone for this group (Figure 2).

### 3.5 Functional Roles and Nesting Patterns of Main Bacterial Taxa Change in Response to Both Selective Pressures

The evaluation of within‐module (Zi) and among‐module (Pi) connectivity revealed similar distributions of nodes across the four conditions, with most nodes as peripheral and no network hubs identified (Figure 3A–D). Interestingly, only one taxon, *Prevotella*, was identified as a module hub in the Ma− network (Figure 3C). Notably, this genus was classified as a connector in CTR− (Figure 3A), whereas in CTR+ and Ma+ (where it was considered a keystone taxon) it was positioned as peripheral (Figure 3B,D). Only one connector was identified in CTR+ (Figure 3B), whereas a higher number of connectors was observed in Ma+ (Figure 3D). An important pattern emerged across conditions: a higher number of connectors was observed in CTR− and Ma+, both characterized by lower community fragmentation (Figure 3A,D).

**FIGURE 3:**
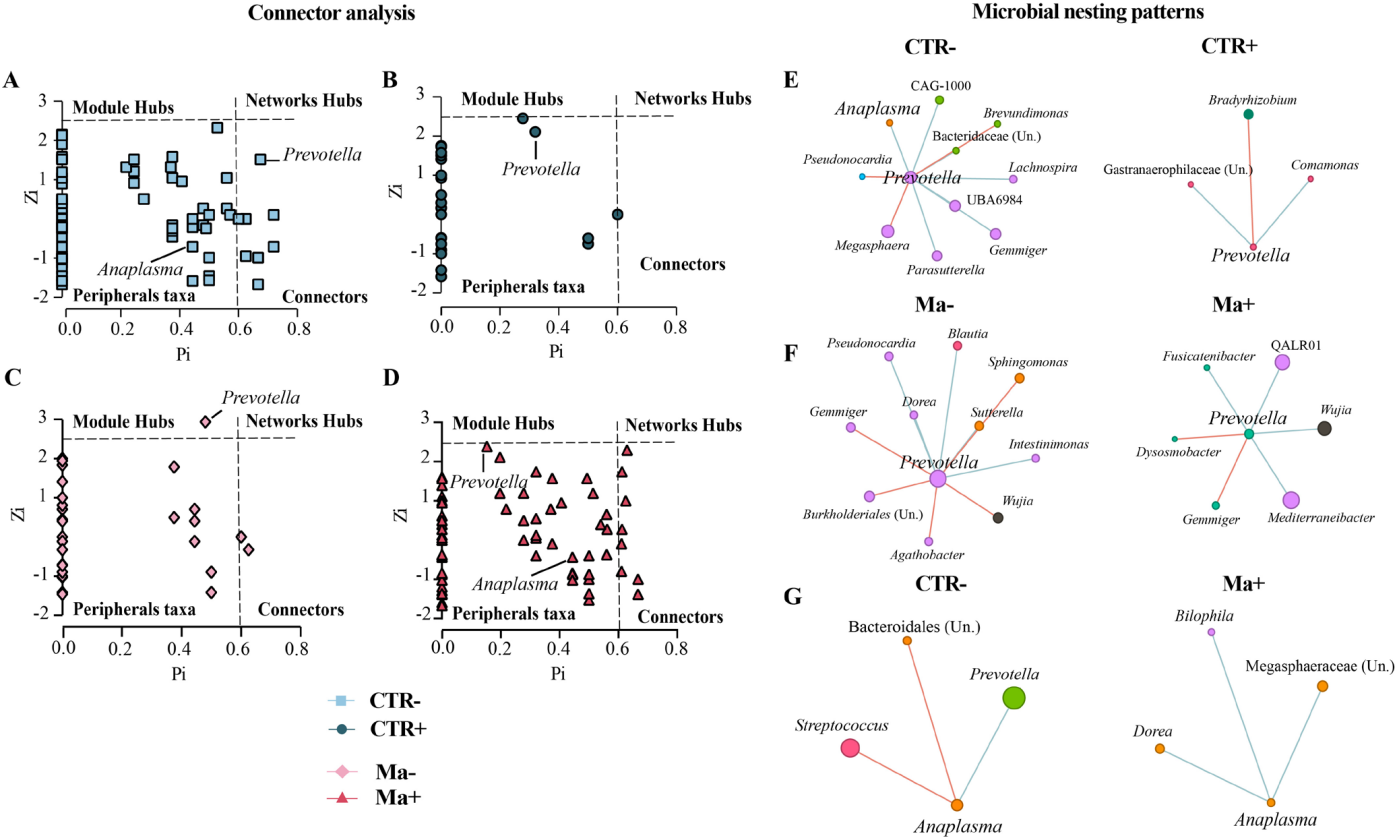
Functional roles and nesting patterns of main bacterial taxa directly influencing the global assembly of the *Rhipicephalus microplus* gut microbiome, as shaped by exposure to *Metarhizium anisopliae* and the presence or absence of *Anaplasma marginale* infection. Centrality measure distribution, within‐module and among‐module connectivities, Zi‐Pi plot of the individual genera from: (A) CTR−, (B) CTR+, (C) Ma− and (D) Ma+. Nesting patterns of *Prevotella* in (E) CTR− and CTR+ and (F) Ma− and Ma+ groups. (G) Directly connected node to *Anaplasma* in CTR− and Ma+. The colours of the edges represent strong positive (red) or negative (blue) correlations (SparCC > 0.75 or < −0.75).

Given its identification as a modular hub in Ma−, a connector in CTR− and a keystone taxon in Ma+, *Prevotella* was selected for further analysis of its nested patterns. In CTR−, *Prevotella* was connected to multiple taxa, whereas in CTR+ its connections involved three taxa (Figure 3E). In Ma−, *Prevotella* also showed multiple connections, whereas in Ma+ it was connected to six taxa (Figure 3F).

In CTR−, *Prevotella* established direct negative associations with the genus *Anaplasma*. This pattern suggests a broader ecological incompatibility between *Prevotella* and members of the genus *Anaplasma*, not limited to *A. marginale* alone. Based on these patterns, the nesting of *Anaplasma* in CTR− (basal community) and Ma+ (dual selective pressure) was further analysed (Figure 3G). In the CTR− network, in addition to the negative association with *Prevotella*, the genus *Anaplasma* (species other than *A. marginale* ) established three direct interactions: two positive associations with *Streptococcus* and Bacteroidales (Un.) (Figure 3G). On the other hand, under dual pressure (Ma+), *Anaplasma* exhibited exclusively negative interactions with all taxa to which it was connected.

### 3.6 Bacterial Community Stability in the Gut of *R. microplus* Responds Differently to *A. marginale* Infection and Early‐Stage From *M. anisopliae* Exposure

To explore how *R. microplus* gut microbial communities respond to biotic stresses, the stability of bacterial interaction networks was evaluated under conditions of *A. marginale* infection and early to *M. anisopliae* exposure. The robustness assessment demonstrated that the CTR− network exhibited a more gradual loss of connectivity under different node removal scenarios compared to CTR+ (Figure 4). In the robustness analysis, the CTR+ network lost approximately 80% of its connectivity remarkably faster than the other treatments, regardless of the node removal method applied (Figure 4).

**FIGURE 4:**
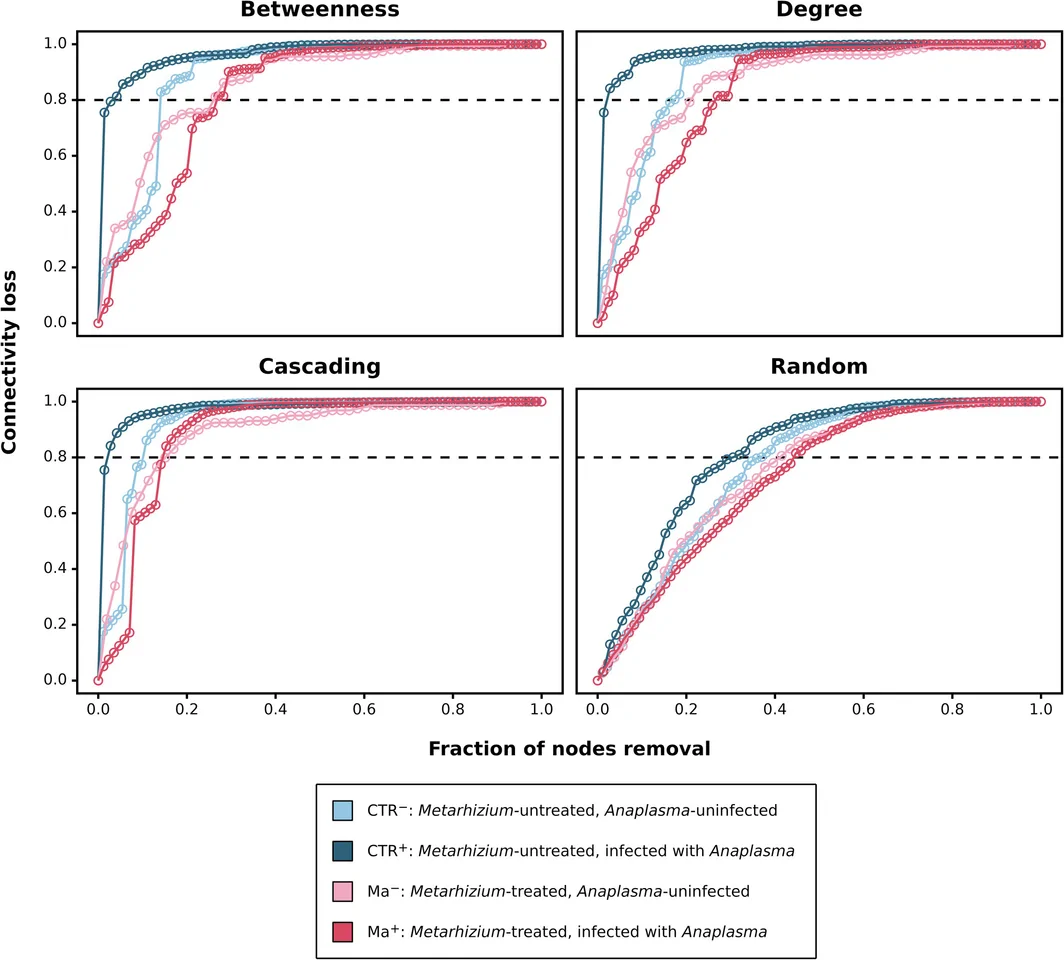
Robustness analysis of bacterial co‐occurrence networks to assess the influence of early ecological interactions on community stability under taxon loss. Graphs representing the loss of connectivity of the co‐occurrence networks of each treatment under different node removal scenarios: Targeted attack removing nodes with the highest Betweenness Centrality and linkage degrees (top), cascade effect with sequential removal and recalculation of Betweenness Centrality (bottom left) and random removal of taxa (bottom right). Comparisons were performed between CTR+ vs. CTR− and Ma+ vs. Ma− for *A. marginale* infection, CTR+ vs. Ma+ and CTR− vs. Ma− for *M. anisopliae* exposure, evaluated at a 0.8 connectivity loss threshold.

The Ma− network showed a progressive loss of connectivity, maintaining a substantial level of interactions even after the removal of a considerable fraction of its nodes. The Ma+ network revealed a remarkably similar robustness pattern to Ma− (Figure 4). Both *Metarhizium*‐treated networks showed a greater ability to preserve network connectivity after node removal than the CTR networks (Figure 4).

To assess how bacterial community networks respond to structural perturbations, their robustness was evaluated under a node addition simulation. Pairwise comparisons were designed to specifically evaluate the effects of *A. marginale* infection (Figure 5A–D) and *M. anisopliae* exposure (Figure 5E–H). Analysis of connectivity metrics revealed consistent differences between treatments. The CTR− network had higher LCC values than the CTR+ network, indicating that the absence of *A. marginale* infection favoured a more cohesive and robust community structure (Figure 5A). In contrast, the CTR+ network showed higher APL values than the CTR− network (Figure 5C), suggesting that *A. marginale* infection reduced network efficiency by increasing the number of steps required for interactions between taxa.

**FIGURE 5:**
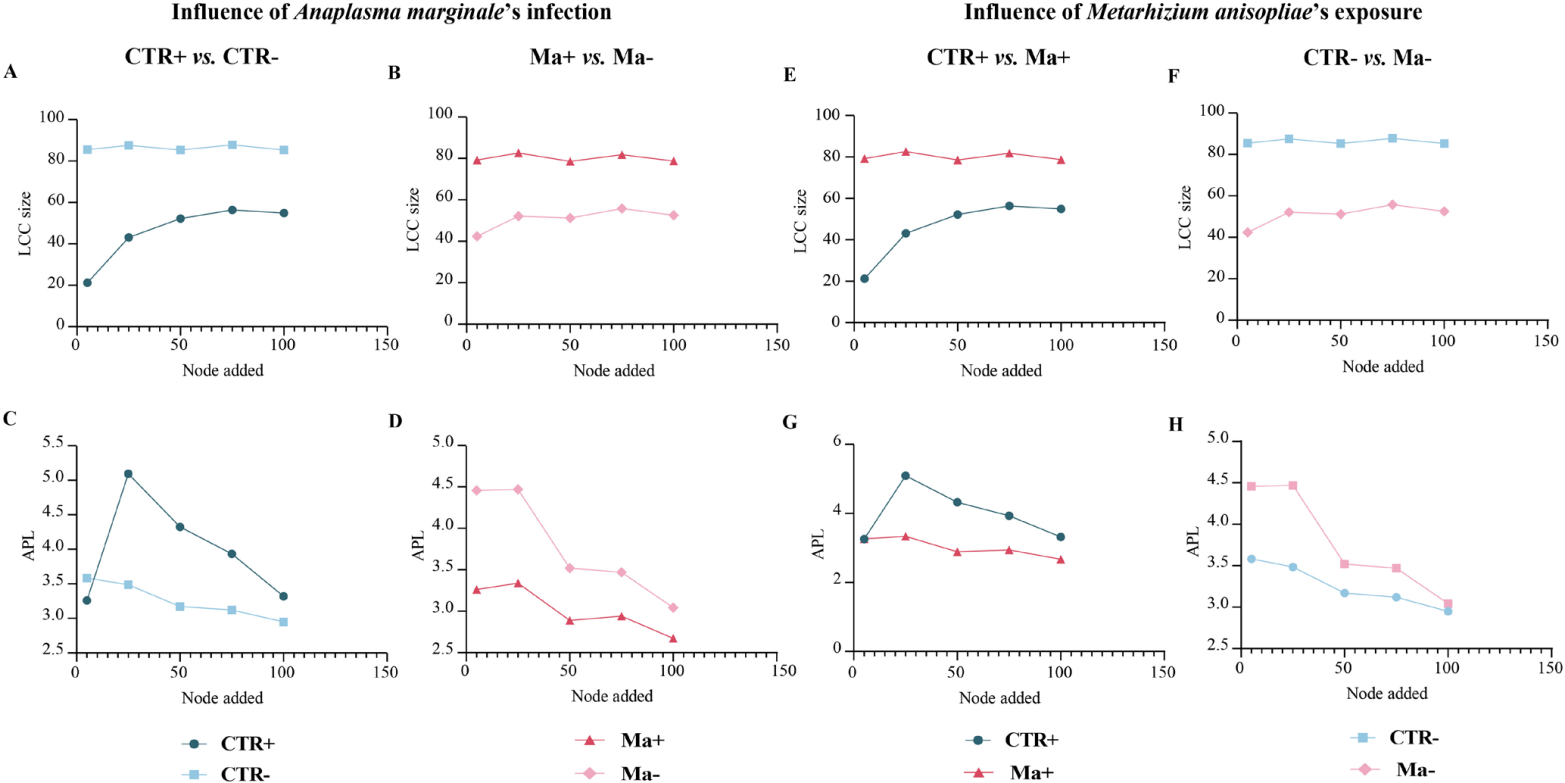
Effect of early ecological interactions driven by *Metarhizium anisopliae* exposure *Anaplasma marginale* infection on microbiome network robustness under node addition. New nodes were randomly connected to the existing network and changes were quantified by evaluating the Largest Connected Component (LCC) size and Average Path Length (APL). Panels show: *A. marginale* infection—(A) CTR− vs. CTR+ LCC, (B) Ma− vs. Ma+ LCC, (C) CTR− vs. CTR+ APL, (D) Ma− vs. Ma+ APL; *M. anisopliae* colonization—(E) CTR+ vs. Ma+ LCC, (F) CTR− vs. Ma− LCC, (G) CTR+ vs. Ma+ APL and (H) CTR− vs. Ma− APL.

For groups exposed to *Metarhizium*, the Ma+ network had higher LCC values than the Ma− network, reflecting a greater ability to maintain connectivity under exposure to the fungus combined with *A. marginale* infection (Figure 5B). In contrast, the Ma− network had higher APL than the Ma+ network, indicating that the community exposed only to the fungus required longer routes for interactions, while the dual condition (Ma+) promoted shorter and more efficient connections (Figure 5D).

The Ma+ network had higher LCC values than CTR+, indicating that the combination of *M. anisopliae* exposure and *A. marginale* infection favoured greater network robustness (Figure 5E). In contrast, CTR+ showed higher APL values than Ma+ (Figure 5G), indicating longer average paths between connected taxa in CTR+ than in Ma+.

Similarly, CTR− had higher LCC values than Ma−, reflecting that the unexposed, uninfected community maintained greater robustness compared to the community exposed only to *M. anisopliae* (Figure 5F). However, Ma− had higher APL values than CTR−, indicating that fungal exposure in the absence of *A. marginale* infection introduced inefficiencies into the network by lengthening interaction paths (Figure 5H).

### 3.7 Bacterial Community Functional Profiles in the Gut of *R. microplus* Respond Differently to *A. marginale* Infection and Early‐Stage From *M. anisopliae* Exposure

The results did not show distinct grouping of KEGG classes among the treatments (Figure 6A). The most abundant functional classes in relative terms were Carbohydrate Metabolism (13.17%), Amino Acid Metabolism (11.83%) and Cofactor and Vitamin Metabolism (9.63%), reflecting essential metabolic pathways prevalent in all samples. Functional diversity analysis revealed that the CTR− treatment had a significantly lower mean diversity than the other treatments (Figure 6B).

**FIGURE 6:**
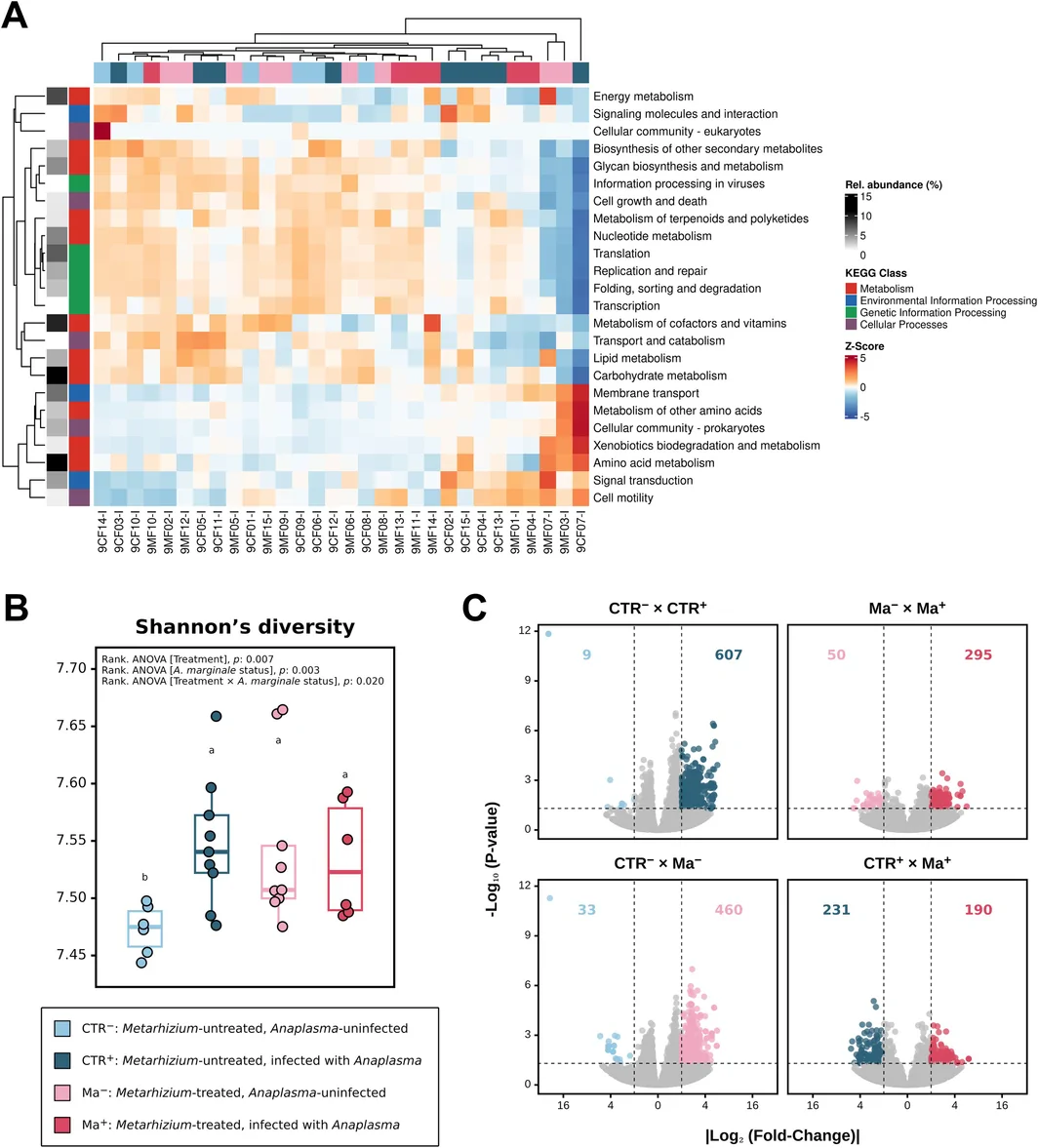
Predicted functional profiles of KEGG orthologs across the treatments. (A) Heatmap showing the distribution of KEGG orthologs (KOs) categorized into subclasses of the KEGG classes ‘Metabolism’, ‘Environmental information processing’, ‘Genetic information processing’ and ‘Cellular processes’. Abundances were transformed into *Z*‐scores, with the overall abundance and KEGG class indicated in the left column. Sample treatments are represented by colour‐coded bars above the heat map. Hierarchical clustering of the rows and columns was performed using the Ward method. (B) Boxplots of functional diversity inferred from the Shannon diversity of KOs across treatments. Statistical analysis was conducted using two‐way ANOVA on ranks (*p* < 0.05), with group differences indicated by different letters based on Fisher's least significant difference (LSD) post hoc test. (C) Volcano plots showing differential abundance analysis of orthologs with thresholds of *p* < 0.05 and |Log_2_ fold‐change| > 1. The annotated numbers indicate the number of the most abundant KOs in each treatment.

In the differential abundance analysis of functions (Figure 6C), volcano plots showed that the groups untreated or treated with *M. anisopliae* infected with *A. marginale* (CTR+ and Ma+) had a higher number of abundant features. In contrast, in treatments without *A. marginale* infection (CTR− vs. Ma−), the presence of *M. anisopliae* (Ma−) led to a considerable increase in the number of more abundant features. However, in treatments with *A. marginale* infection (CTR+ vs. Ma+), an opposite trend was observed, with CTR+ showing more abundant features than Ma+. The predicted functional profiles should be interpreted as potential functional shifts rather than direct evidence of metabolic alterations. Further multi‐omics studies will be necessary to confirm the functional implications suggested by the present results.

### 3.8 Bacterial Community Assembly and Stability in the Gut of *R. microplus* Respond Differently to the Basal Absence and Removal of *A. marginale* Under Exposure to *M. anisopliae*

To determine the ecological effects of *A. marginale* on gut bacterial communities exposed to *M. anisopliae*, we compared the assembly patterns and stability of the Ma+ network following *in silico Anaplasma's* removal (Ma + woA) with those observed in Ma−, where the pathogen was absent from the outset (Figure 7).

**FIGURE 7:**
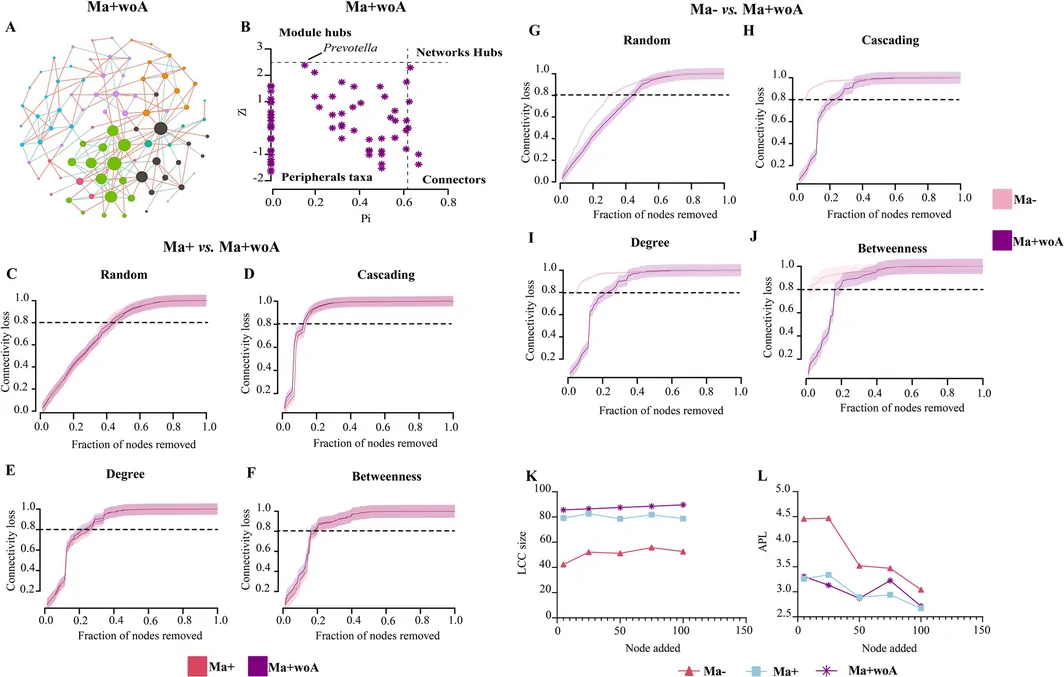
Microbial community assembly and stability after removal of the genus *Anaplasma* within a context of incipient ecological interactions. (A) Global co‐occurrence networks after *Anaplasma*'s removal (Ma + woA). Node colours are based on modularity class metric and equal colour means modules of co‐occurring taxa. The size of the nodes is proportional to the eigenvector centrality of each taxon. (B) Centrality measure distribution, within‐module and among‐module connectivities, Zi‐Pi plot of the individual genera from Ma + woA. Robustness comparison between Ma+ vs. Ma + woA networks after (C) random and directed attacks in (D) cascading, (E) degree and (F) betweenness. Robustness comparison between Ma− vs. Ma + woA networks after (G) random and directed attacks in (H) cascading, (I) degree and (J) betweenness. Robustness analysis during node‐addition scenarios. New nodes were randomly connected to the existing network and changes in stability were quantified by measuring the (K) LCC and the (L) APL values.

The *in silico* removal of *A. marginale* from the Ma+ network (Ma + woA) resulted in a higher number of interacting nodes (87) and total interactions (201) compared to the other conditions (Figure 7A). The interaction profile of Ma + woA was more similar to Ma+, with a predominance of negative over positive associations, than to Ma−.

In contrast, analysis of modularity revealed a different pattern. The Ma + woA network displayed a high modularity value (0.599), resembling the structure of Ma− rather than Ma+ (Figure 7A). The modularity values were consistent with the number of connectors observed across networks. The Ma + woA network was more fragmented than Ma+, which was reflected in a lower number of connectors (Figure 7B). Conversely, Ma + woA was less fragmented than Ma−, showing a comparatively higher number of connectors (Figure 7B). In addition, the removal of *Anaplasma* did not alter the functional role of *Prevotella*, which remained a peripheral taxon (Figure 7B). This finding is consistent with the interaction observed in the original Ma+ network, in which *Prevotella* and *Anaplasma* did not maintain direct interactions (Figure 3G).

In terms of robustness, the Ma+ and Ma + woA networks exhibited a similar behaviour, indicating that the in silico removal of *Anaplasma* did not substantially alter the overall resistance of the community to taxon removal (Figure 7C–F). This similarity was observed under random and cascading node removal (Figure 7C,D, respectively), as well as under degree‐ and betweenness‐based removal strategies (Figure 7E,F, respectively).

In contrast, a considerable difference was observed between Ma− and Ma + woA (Figure 7G–J), reinforcing the idea that the elimination of the pathogen does not restore the community to a basal state equivalent to one that has never been subjected to pathogen‐driven selective pressure. These comparisons included random removal (Figure 7G), cascading removal (Figure 7H) and removal based on degree and betweenness (Figure 7I,J, respectively). This supports the notion that the presence of *A. marginale* leaves a lasting ecological imprint on bacterial community organization.

Interestingly, the Ma + woA network was more robust than Ma+, which is consistent with its lower degree of fragmentation. Regarding network addition, Ma + woA also displayed a behaviour more similar to Ma+ than to Ma− (Figure 7K,L). Specifically, the LCC values of Ma + woA were closer to those of Ma+ than to Ma− (Figure 7K), whereas APL values varied across the three conditions, with Ma− generally showing higher values than Ma+ and Ma + woA (Figure 7L).

## 4 Discussion

Entomopathogenic fungi have been widely explored for the biological control of *R. microplus* , an important vector of *A. marginale* (Beys‐da‐Silva et al. 2020; Hong et al. 2023; Klafke et al. 2024; Rodrigues et al. 2019; Ferreira et al. 2022). Although *M. anisopliae* can reach the tick gut within 72 h (Arruda et al. 2005; Mesquita et al. 2020), fungal DNA was not consistently detected, indicating that exposure does not necessarily result in intestinal colonization. However, early fungal–host interactions can trigger physiological and immune responses before colonization becomes detectable (Schrank and Vainstein 2010; Liu et al. 2023; Kurtti and Keyhani 2008). Therefore, ‘fungal treatment’ in this study refers to fungal exposure rather than confirmed gut colonization, as exposure alone was sufficient to initiate ecological interactions within the tick bacteriome.

The tick microbiota is shaped by both intrinsic and extrinsic factors, including host‐derived microorganisms and environmental influences (Bonnet et al. 2017; Narasimhan and Fikrig 2017; Piloto‐Sardiñas et al. 2024). To minimize host‐associated variation, all ticks in this study fed on the same naturally infected *A. marginale* ‐positive bovine, providing a more controlled framework to evaluate microbial responses to fungal exposure and pathogen infection. Despite this standardization, qPCR revealed considerable variation in *A. marginale* loads among individual ticks' related factors, indicating that intrinsic host‐related factors continue to influence pathogen acquisition and bacteriome organization. This heterogeneity reduced the effective sample size of infection‐based comparisons and may have limited the detection of subtle effects. Nevertheless, the use of a single host represented an intentional trade‐off, reducing variation in blood composition and microbial background while allowing a more consistent assessment of tick‐level ecological responses.

Despite the absence of diversity shifts, functional and compositional differences were detected among treatments. Differential abundance analysis showed that while CTR− and Ma+ harboured more associated taxa, CTR+ and Ma− exhibited simplified profiles with fewer biomarkers. Although the basal bacteriome revealed only biomarkers classified as environmental bacteria, this does not imply the absence of natural endosymbionts, but rather that environmental taxa, more variable and adaptable, became statistically more predominant. Interestingly, Ma+ harboured taxa adapted to challenging environments, such as *A. muciniphila* , *Paramuribaculum* and *Pantoea dispersa* , suggesting that combined fungal and pathogen pressure may create unique ecological conditions that reshape the bacteriome. The predominance of environmental bacteria across groups, including taxa like *P. dispersa* , reinforces the idea that transient or environmentally derived microbes can play functional roles in tick physiology, influencing immunity, metabolism and pathogen defence (Bonnet et al. 2017; Maldonado‐Ruiz 2024). Similar patterns have been observed in other arthropods, such as *Aedes aegypti* treated with *Beauveria bassiana*, where environmental bacteria increased at the expense of resident bacteriota (Yin et al. 2025).

Taken together, these findings suggest that fungal exposure and *A. marginale* infection establish an incipient ecological interaction within the first 72 h. Although bacterial diversity and overall community composition remained largely stable, co‐occurrence analyses revealed early network reorganization under selective pressures, particularly under dual exposure (Ma+). The absence of *A. marginale* from the CTR+ network, despite qPCR confirmation, likely reflects its high abundance and weak dependence on other taxa, reducing detectable correlations (Wu‐Chuang et al. 2023). In contrast, the basal community (CTR−) exhibited a fragmented network dominated by negative interactions, consistent with competitive assembly patterns reported for communities lacking strong external selection (Mateos‐Hernández et al. 2020; Paulino et al. 2024). Dual pressure (Ma+) intensified these negative associations while increasing connectivity, suggesting that pathogen history and fungal exposure jointly reshape bacterial interactions before detectable shifts in diversity and composition emerge.

The robustness analyses revealed that *A. marginale* infection alone (CTR+) was associated with reduced robustness of the gut microbial network of *R. microplus* , accelerating connectivity loss and reducing efficiency, consistent with the idea that pathogen colonization exploits niches and increases community vulnerability (Piloto‐Sardiñas et al. 2024). In contrast, fungal exposure alone (Ma−) was associated with greater fragmentation and longer interaction paths, suggesting early microbial network reorganization following that *M. anisopliae* triggers an incipient competitive state that reorganizes the community but at the cost of efficiency. Unexpectedly, the dual condition (Ma+) did not exhibit further reductions in network cohesion and instead exhibited a comparatively more connected network configuration than infection alone.

Although multiple selective pressures are generally expected to increase community susceptibility, the dual‐pressure condition (Ma+) exhibited a more cohesive network configuration than infection alone. This suggests that prior microbial restructuring may shape responses to subsequent stressors. Consistent with other ecosystems, basal communities (CTR−) showed fragmented and competitive structures, whereas selective pressures promoted network reorganization and potential adaptive restructuring (Mateos‐Hernández et al. 2020; Paulino et al. 2024).

Taken together, these findings indicate that while *A. marginale* alone was associated with greater sensitivity to ecological modifications, such as taxon loss or invasion (Piloto‐Sardiñas et al. 2024), the additional pressure of fungal exposure may contribute to a more stable community organization. This dual effect underscores the importance of considering combined biotic stresses when evaluating microbiome resilience and highlights the potential of entomopathogenic fungi not only as direct control agents but also as modulators of microbial community stability.

Our results demonstrate that the *in silico* removal of *A. marginale* destabilizes the gut bacterial community of *R. microplus* . This finding is consistent with the observations of Piloto‐Sardiñas et al. (2024), who reported that the presence of *Anaplasma* reduces biodiversity and increases microbial sensitivity to ecological processes such as taxon loss and invasion. Importantly, our data extend this view by showing that the behaviour of the community after pathogen removal differs markedly from that of a basal community (Ma−) that has never been subjected to pathogen‐driven selection.

The Ma + woA network retained structural and functional signatures of prior colonization, with interaction patterns more similar to Ma+ than to Ma−, reinforcing the idea that *A. marginale* leaves a lasting persistent association pattern on community assembly. This imprint was evident in the persistence of negative associations and in the fact that removal did not restore the network to a naïve state, but rather generated a reorganized structure with intermediate fragmentation and robustness. Thus, the ecological history of pathogen colonization shapes subsequent community responses, highlighting that displacement of *A. marginale* does not erase its influence but instead redefines the stability and assembly rules of the gut bacteriome in *R. microplus* .

## 5 Conclusions

Our findings suggest that *R. microplus* gut bacteriota responds in complex and context‐dependent ways to pathogen infection and fungal exposure. While *A. marginale* alone destabilizes microbial networks and reduces resilience, the additional pressure of *M. anisopliae* exposure paradoxically promotes reorganization and may promote a more stable microbial organization, even under dual stress. The persistence of inferred functional patterns after pathogen removal underscores that *A. marginale* leaves a lasting ecological footprint on community assembly, shaping subsequent responses to biotic pressures. At the same time, the ability of *M. anisopliae* to be associated with more connected and potentially stable microbial interaction patterns, without compromising its pathogenic action, highlights its dual role as both a direct biocontrol agent and an indirect modulator of microbiome resilience. Together, these results provide novel ecological insights and reinforce the potential of entomopathogenic fungi as sustainable tools for integrated tick management and the mitigation of vector‐borne diseases in livestock systems.

## Author Contributions

**Thaís Almeida Correa:** conceptualization, methodology, investigation, project administration, writing – original draft, writing – review and editing. **Elianne Piloto‐Sardiñas:** methodology, formal analysis, writing – original draft, writing – review and editing. **Lucas Amoroso Lopes de Carvalho:** formal analysis, writing – review and editing. **Patrícia Gonzaga Paulino:** investigation, formal analysis, writing – review and editing. **Daniel Guariz Pinheiro:** formal analysis, writing – review and editing. **Isabele da Costa Angelo:** funding acquisition, resources, writing – review and editing. **Vânia R. E. P. Bittencourt:** funding acquisition, resources, writing – review and editing. **Alejandro Cabezas‐Cruz:** methodology, writing – review and editing. **Huarrisson Azevedo Santos:** conceptualization, methodology, funding acquisition, resources, writing – review and editing. **Patrícia Silva Golo:** conceptualization, methodology, supervision, resources, funding acquisition, writing – original draft, writing – review and editing.

## Funding

This work was supported by the Coordenacão de Aperfeiçoamento de Pessoal de Nível Superior (CAPES) (Finance Code 001), the Fundação Carlos Chagas Filho de Amparo à Pesquisa do Estado do Rio de Janeiro (E‐26/204.509/2024 and E‐26/201.644/2025) and the Conselho Nacional de Desenvolvimento Científico e Tecnológico CNPq/INCT Innovative Bioinputs (406803/2022‐6).

## Ethics Statement

This study was carried out in accordance with the Project approved by the Ethics Committee for the Use of Animals (CEUA) of the Veterinary Institute, Rio de Janeiro Federal Rural University (UFRRJ), Seropédica, Brazil (protocol no. 4832020623/approval date: 02/10/2023 and protocol no. 6274250624/approval date: 02/09/2024). As the present study accessed Brazilian genetic heritage, the research was registered at the National System for the Management of Genetic Heritage and Associated Traditional Knowledge (Sisgen) under the code AA47CB6.

## Conflicts of Interest

The authors declare no conflicts of interest.

## Supporting information


**Data S1:** Supplementary Methods.
**Data S1:1** Calf selection and tick collection.
**Data S1:2** *Metarhizium anisopliae* fungal suspension.
**Data S1:3** Analysis of the DNA quality and presence of *A. marginale* in the experimental groups.
**Data S1:4** Detection of *M. anisopliae* in the gut of *R. microplus* female.
**Table S1:** Primers and probes sequences used for quantitative PCR analyses.
**Data S1:5** Processing of raw reads.
**Data S1:6** Microbiome analysis.
**Data S2:** Supplementary Results.
**Data S2:1** The gut bacteriome of *R. microplus* maintains bacterial community diversity and structure under *A. marginale* infection and early‐stage from *M. anisopliae* exposure.
**Figure S1:** Influence of early ecological interactions arising from exposure to *Metarhizium anisopliae*, in the absence and presence of *Anaplasma marginale* infection, on the diversity and composition (shared and unique ASVs) of the bacterial community associated with *Rhipicephalus microplus* . (A) Venn diagram displaying the distribution of shared and unique ASVs among treatment groups. (B) Microbial composition among samples by principal coordinate analysis (PCoA) of weighted UniFrac distances. Statistical significance was assessed using a two‐way PERMANOVA (*p* < 0.05). Small circles represent the samples and ellipses represent the dispersion of the samples. (C) Alpha diversity metrics, including ASV richness, Pielou's evenness and Faith's phylogenetic diversity (Faith's PD), were compared using a two‐way ANOVA on ranks (*p* < 0.05).
**Figure S2:** Beta dispersion analysis of principal coordinates (PCoA) of weighted UniFrac distances illustrating differences in microbial composition between samples. Statistical significance was assessed using two‐way ANOVA (*p* < 0.05).
**Data S2:2** Shared taxa predominate in the gut bacteriome of *R. microplus* across experimental groups treated with *M. anisopliae* and infected with *A. marginale* .
**Figure S3:** Gut bacterial taxonomic composition and abundance in *Rhipicephalus microplus* shaped by early ecological interactions arising from exposure to *Metarhizium anisopliae*, assessed under conditions with and without *Anaplasma marginale* infection. (A) Venn diagrams illustrating the overlap of bacterial phyla and genera between the treatment groups (CTR−, CTR+, Ma− and Ma+). (B) Bar graphs showing the relative abundance of bacterial phyla in the treatments. Low‐abundance phyla were grouped under ‘Others’. (C) Heatmap displaying the distribution of the 50 most abundant bacterial genera, with abundance transformed into *Z*‐scores. The overall abundance and corresponding phyla are indicated in the left column of the graph. Sample treatments are represented by colour‐coded bars above the graph. Hierarchical clustering of rows and columns was performed using Ward's method.
**Figure S4:** Relative abundance of bacterial families in *Rhipicephalus microplus* guts, comparing samples treated or untreated with *Metarhizium anisopliae* and infected or uninfected with *Anaplasma marginale* . Low‐abundance families are grouped under ‘Others’.

## Data Availability

The datasets generated for this study were deposited in the NCBI—Sequence Read Archive (SRA), under the accession number PRJNA1259439.
