# Supplementary material for: Gut Bacteriome Modulation in Rhipicephalus microplus Under Biotic Stress From Metarhizium anisopliae and Anaplasma marginale
Source: Environ Microbiol Rep. 2026 Nov 10;18(6):e70455. doi: 10.1111/1758-2229.70455 (PMC13751325; doi:10.1111/1758-2229.70455)
Supplement: Supplementary file 1 [file EMI4-18-e70455-s001.docx]

Environmental Microbiology Reports

Gut bacteriome modulation in Rhipicephalus microplus under biotic stress from Metarhizium anisopliae and Anaplasma marginale

Thaís Almeida Correa^1^, Elianne Piloto-Sardiñas^2^, Lucas Amoroso Lopes de Carvalho^3^, Patrícia Gonzaga Paulino^4^, Daniel Guariz Pinheiro^5^, Isabele da Costa Angelo^4^, Vânia R.E.P. Bittencourt^6^, Alejandro Cabezas-Cruz^7^, Huarrisson Azevedo Santos^4^, Patrícia Silva Golo^6^*****

***Corresponding author:** Patrícia Silva Golo ([patriciagolo@gmail.com](mailto:patriciagolo@gmail.com))

Supporting Information

**S1. Supplementary Methods**

**S1.1 Calf selection and tick collection**

Blood samples were collected from the jugular vein using sterile Vacutainer needles and EDTA-coated tubes (Becton Dickinson, Curitiba, Brazil), for molecular confirmation of *A. marginale* infection. Samples were stored at 4 °C at the W.O. Neitz Parasitological Research Station, Laboratory of Microbial Control of Arthropods of Veterinary Importance (LCM) at the Federal Rural University of Rio de Janeiro (UFRRJ), Brazil. DNA was extracted using the DNeasy Blood & Tissue Kit (QIAGEN, São Paulo, Brazil), according to the manufacturer's instructions and used for detection by Nested PCR (nPCR) according to de Echaide et al. (1998). R. microplus larvae from the Porto Alegre strain colony, maintained at the LCM under controlled laboratory conditions, were used for artificial infestation of the A. marginale-positive calf.

**S1.2 *Metarhizium anisopliae* fungal suspension**

M. anisopliae LCM S04 was cultured on oat medium (60 g oat bran and 12.5 g agar in 1000 mL distilled water) under controlled conditions (25 ± 1 °C and RH ≥ 80%). Conidial viability was assessed by plating a 20 μL aliquot of a 1 × 10⁵ conidia mL⁻¹ suspension on potato dextrose agar (PDA), followed by incubation at 25 ± 1 °C and RH ≥ 80%. After 24 h, conidial germination was evaluated by optical microscopy (400×) (Nikon E200, Tokyo, Japan) after staining with methyl blue (Sigma-Aldrich, St. Louis, MO, USA). A minimum of 300 conidia were examined, including germinated and non-germinated conidia, and the germination percentage was calculated according to Lopes et al. (2013). Conidia were considered germinated when visible germ tubes were observed.

**S1.3 Analysis of the DNA quality and presence of *A. marginale* in the experimental groups**

Detection of A. marginale DNA was performed by qPCR targeting the msp1β gene according to Carelli et al. (2007) (Table S1). Reactions were performed in triplicate using the StepOnePlus™ Real-Time PCR System (Applied Biosystems, Thermo Fisher Scientific, Waltham, MA, USA), with a final reaction volume of 12 µL containing 6 µL of TaqMan Universal PCR Master Mix, 0.5 µM of each primer, 0.25 µM of probe, and 3 µL of total DNA (30 ng/µL). Molecular-grade water was used as the negative control, while DNA extracted from bovine blood naturally infected with A. marginale was used as the positive control.

Thermocycling conditions consisted of 50 °C for 2 min, 95 °C for 10 min, followed by 40 cycles of 95 °C for 30 s and 72 °C for 30 s. For normalization, the endogenous *R. microplus* 18S rRNA gene was amplified according to Saldivar et al. (2008) (Table S1). Reactions were conducted in triplicate in a final volume of 12 µL containing 6 µL of TaqMan Universal PCR Master Mix, 0.9 µM primer, 0.25 µM probe, and 3 µL DNA template. Thermocycling conditions consisted of 95 °C for 10 min, followed by 40 cycles of 95 °C for 15 s and 60 °C for 1 min.

**S1.4 Detection of *M. anisopliae* in the gut of *R. microplus* female**

The presence of *M. anisopliae* DNA in tick gut samples was evaluated by qPCR targeting the elongation factor 1-alpha (*EF-1α*) gene according to Nijhof et al. (2009). Reactions were performed in triplicate using the StepOnePlus™ Real-Time PCR System (Applied Biosystems, Thermo Fisher Scientific, Waltham, MA, USA), with a final volume of 12 µL containing 6 µL of SYBR^®^ PCR Master Mix (Applied Biosystems, Thermo Fisher Scientific, Waltham, MA, USA), 0.5 µM of each primer, and 2.5 µL of total DNA. Molecular-grade water was used as the negative control, while DNA extracted from *M. anisopliae* cultured on oat medium was used as the positive control.

Thermocycling conditions consisted of an initial denaturation step at 95 °C for 10 min, followed by 40 cycles at 95 °C for 20 s, 60 °C for 30 s, and 72 °C for 30 s. Melt curve analysis was subsequently performed using temperature increments of 0.3 °C every 15 s from 60 °C to 95 °C. Primer sequences are described in Table S1.

**Table S1 Primers and probes sequences used for quantitative PCR analyses**

| **Target** | **Primer or probe**^a^ | **Sequence (5’-3’)** |
| --- | --- | --- |
| *Anaplasma marginale (msp* 1β*)* | AM – F | TTGGCAAGGCAGCAGCTT |
|  | AM – R | TTCCGCGAGCATGTGCAT |
|  | AM – Pb | 6 FAM – TCGGTCTAACATCTCCAGGCTTTCAT - QSY |
| *Rhipicephalus microplus* (18S rRNA) | Rm18SF | CCTGAGAAACGGCTACCACATC |
|  | Rm18SR | TGCCGGGAGTGGGTAATT |
|  | Rm – Pb | VIC-AGGAAGGCAGCAGGCGCGC |
| *Metarhizium anisopliae* Elongation Factor 1-alpha (EF-1α) | ELF1A-F | CGTCTACAAGATTGGTGGCATT |
|  | ELF1A-R | CTCAGTGGTCAGGTTGGCAG |

^a^ F: forward primer; R: reverse primer; Pb: probe

**S1.5 Processing of raw reads**

Raw reads were assessed for quality using FastQC software (Andrews, 2010). Primer sequences were trimmed using Atropos (Didion et al., 2017) with the “--discard-untrimmed” option to eliminate unprimed sequences. Reads with an average Phred quality score below 25 were excluded using the “--average_qual 25” parameter. Read pairs were merged using FLASH (Magoc and Salzberg, 2011) with default settings. The resulting high-quality merged reads were then processed using the DADA2 pipeline (Callahan et al., 2016) implemented in R (R Core Team, 2023). The “filterAndTrim” function was applied to remove sequences with more than four expected errors (maxEE = 4). The Amplicon Sequence Variants (ASV) were inferred for each sample, followed by filtering of chimeric sequences using the “removeBimeraDenovo” function to retain biologically correct sequences. The ASVs were taxonomically classified using the “assignTaxonomy” function of DADA2, referencing the “Greengenes2” database (McDonald et al., 2024). Additionally, ASVs were aligned with the NCBI RefSeq 16S rRNA database using BLAST (Altschul et al., 1990) for secondary verification. Phylogenetic relationships among ASVs were inferred using the Neighbor-Joining algorithm with the NJ function in the phangorn package (Schliep, 2011). Statistical support for the phylogenetic tree was assessed using the “bootstrap” method with the “bootstrap.pml” function, employing 100 bootstrap replicates. Finally, potential contaminant sequences were identified and removed using the Decontam R package (Davis et al., 2018). “Blank” samples containing 0.9% NaCl, processed together with the experimental treatments, were included in the sequencing batch to detect contaminants. Decontam was run using the “prevalence” method with a detection limit of 0.5, and identified contaminant ASVs were excluded from further analyses.

**S1.6 Microbiome analysis**

*Microbial diversity and composition*

Sampling efficiency was assessed using rarefaction curves generated with the “amp_rarecurve” function of the R package, ampvis2 (Andersen et al., 2018). The samples were rarefied to the library with the lowest number of sequences (8,868 reads), and all subsequent analyses were performed based on rarefied data. The ASV counts and taxonomic annotations were exported in the phyloseq format (R package, phyloseq; McMurdie and Holmes, 2013). To standardize the dataset for microbiome analyses, the phyloseq object was transformed into compositional data using the “phyloseq_standardize_otu_abundance” function of the R package MetagMisc (Mikryukov, 2022). Alpha diversity was calculated using observed richness measures (Richness) and Pielou's evenness indices, using the “alpha” function of the R package microbiome (Lahti and Shetty, 2012) and Faith's phylogenetic diversity, using the “pd” function of the R package picante (Kembel et al., 2010). Beta diversity was analyzed by UniFrac distances (weighted) between samples using the “distance” function of the phyloseq. The beta dispersion of the samples within each treatment group was also assessed to evaluate the variation between groups using the “betadisper” function of the R package vegan (Oksanen et al., 2019). For biomarker detection, the Linear Discriminant Analysis Effect Size (LEfSe) approach (Segata et al., 2022) was used at each taxonomic level. This analysis was implemented using the MicrobiomeAnalyst web platform (Dhariwal et al., 2017), and the thresholds for significance were *p* < 0.05 and LDA > 1.5.

*Microbial co-occurrence networks*

Co-occurrence network analyses were performed using the “Sparse Correlations for Compositional Data” (SparCC) method implemented through the R package SpiecEasi (Kurtz et al., 2015). Taxonomic data tables were collapsed to the taxonomic genus level and used to calculate the correlation matrix. Only relationships with correlation coefficients greater than 0.75 or less than ‐0.75 and with a *p*-value ≤ 0.05 were considered. Furthermore, to reduce noise and focus on relevant genera, only those with a summed relative abundance greater than 0.01% in the dataset were included. Co-occurrence networks were constructed and topological characteristics, such as degree of connection, average closeness, and betweenness centrality, were calculated using the R package igraph (Csardi and Nepusz, 2006).

*Local connectivity patterns of main taxa in the microbial community*

To describe local connectivity patterns within the microbial community, the direct relationships of the main taxa identified in the networks were examined, whose assembly patterns could alter community structure and behavior under the experimental conditions. Subnetworks were constructed to visualize each selected taxon along with its direct associations, both positive and negative. Analyses were performed in Gephi v0.10 (Bastian et al., 2009), with edge strength represented by SparCC weights.

*Centrality measures distribution analysis*

To determine the influence of *A. marginale* infection and fungal exposure/colonization with *M. anisopliae* on the contribution of nodes to network connectivity and interactions, the distribution of centrality measures was analyzed in four experimental groups: CTR+, CTR−, Ma+, and Ma−. The topology of the taxa in the network revealed two types of connectivity: (i) within-module connectivity (Zi) and (ii) between-module connectivity (Pi) (Guimerà et al., 2005). Based on these values, taxa were classified into four categories: (i) peripheral taxa (Zi ≤ 2.5 and Pi ≤ 0.62), with few edges inside and outside their module; (ii) connectors (Zi ≤ 2.5 and Pi > 0.62), taxa connected to modules other than their own; (iii) module hubs (Zi > 2.5 and Pi ≤ 0.62), highly connected to members of their own module; and (iv) network hubs (Zi > 2.5 and Pi > 0.62), highly connected to members both within and between modules. For each taxon, Zi and Pi values were calculated using only positive edges, employing the R package “code-zi-pi-plot”, as described by Cao et al. (2018) and Guo et al. (2022) in RStudio (RStudio Team, 2020), and visualized with GraphPad Prism 10 (GraphPad Software, USA). Plots were constructed to allow pairwise comparisons between experimental groups.

*Network robustness analysis in nodes removal and addition*

The robustness of the microbial co-occurrence networks was assessed to determine the influence of *A. marginale* infection and *M. anisopliae* exposure on network stability and resilience in response to node perturbations. Robustness was evaluated under two types of perturbations: node removal and node addition. For node removal, an attack tolerance test was conducted using Network Strengths and Weaknesses Analysis (NetSwan) (Lhomme et al., 2015) in R v.4.0.3 (R Core Team, 2023). Networks were subjected to both random and directed attacks. Directed removal was performed under three scenarios: (i) removal of nodes in decreasing order of betweenness centrality (BNC), (ii) cascading removal with BNC recalculated after each deletion, and (iii) removal based on highest degree centrality. In this analysis, group comparisons were made at the point where 80 % connectivity loss was reached.

For node addition, new nodes were randomly connected to the existing network, and changes were quantified by evaluating the Largest Connected Component (LCC) size and Average Path Length (APL) (Freitas et al., 2021). Nodes were added incrementally in sections from 5 to 100. Wilcoxon signed-rank tests were used to calculate *p*-values for LCC and APL, with Benjamini–Hochberg (BH) adjustment to control the false discovery rate. Bootstrapping was applied to obtain confidence intervals, and significance was set at *p* < 0.05. Pairwise comparisons were therefore performed to specifically evaluate the effect of *A. marginale* infection (CTR+ *vs.* CTR- and Ma+ *vs.* Ma-) and *M. anisopliae* exposure (CTR+ *vs.* Ma+ and CTR- *vs.* Ma-). The values were recorded during the addition of 5 and 100 nodes and the results were visualized in GraphPad Prism 10 (GraphPad Software, USA).

*Functional prediction*

To infer the functional potential of the microbiomes, the PICRUSt2 program (Douglas et al., 2019) was used to predict the KEGG orthologs (KOs) in each sample from the ASVs. Using the predicted orthologs, functional richness and diversity were calculated using the “alpha” function of the Phyloseq package. Differentially abundant functions between conditions were identified using the DESeq2 methodology (R package DESeq2; Love et al., 2014), with significance thresholds set at *p* < 0.05 and |Log2 fold-change| > 1.

**S2. Supplementary Results**

**S2.1 The gut bacteriome of *R. microplus* maintains bacterial community diversity and structure under *A. marginale* infection and early-stage from *M. anisopliae* exposure**

The gut bacteriome of *R. microplus* maintains bacterial community diversity and structure under *A. marginale* infection and early-stage from *M. anisopliae* exposure. Analysis of the gut bacterial composition of all tick samples revealed a total of 698 ASVs, of which 197 (28.22%) were common to all groups (Figure S1A). Only a small number of ASVs were unique to specific treatments, ranging from four ASVs (0.57%) in CTR‐ to 28 ASVs (4.01%) in CTR+, 24 ASVs (3.43%) in Ma‐, and six ASVs (0.85%) in Ma+. PCoA analysis, based on the weighted UniFrac distance matrix, explained 51.77% of the variance observed in the first two axes. PERMANOVA results indicated no significant differences in the composition of gut microbial communities among the analyzed groups (*p* > 0.05) (Figure S1B). However, beta dispersion analysis revealed significant differences, with the Ma+ group showing less dispersion than the CTR+ group (p = 0.014) (Figure S2). Alpha diversity was assessed using three metrics (Richness, Pielou's evenness, and Faith's phylogenetic diversity). No significant differences in alpha diversity were observed between the treatments (*p* > 0.05) (Figure S1C).

**Figure S1** Influence of early ecological interactions arising from exposure to *Metarhizium anisopliae*, in the absence and presence of *Anaplasma marginale* infection, on the diversity and composition (shared and unique ASVs) of the bacterial community associated with *Rhipicephalus microplus*. (A) Venn diagram displaying the distribution of shared and unique ASVs among treatment groups. (B) Microbial composition among samples by principal coordinate analysis (PCoA) of weighted UniFrac distances. Statistical significance was assessed using a two-way PERMANOVA (*p* < 0.05). Small circles represent the samples, and ellipses represent the dispersion of the samples. (C) Alpha diversity metrics, including ASV richness, Pielou’s evenness, and Faith’s phylogenetic diversity (Faith’s PD), were compared using a two-way ANOVA on ranks (*p* < 0.05).

**Figure S2** Beta dispersion analysis of principal coordinates (PCoA) of weighted UniFrac distances illustrating differences in microbial composition between samples. Statistical significance was assessed using two-way ANOVA (*p* < 0.05).

**S2.2 Shared taxa predominate in the gut bacteriome of *R. microplus* across experimental groups treated with *M. anisopliae* and infected with *A. marginale***

Taxonomic assignment was highly effective, with 98.82% of sequences successfully classified at the genus level. Venn diagrams at the phylum and genus levels (Figure S3A) revealed substantial taxon sharing between the treatments. Of the 12 phyla identified, none were exclusive to a single treatment, and eight phyla (66.6%) were shared across all groups. At the genus level, 125 of the 234 taxa (53.4%) were shared among the groups, whereas only one genus (0.42%) was exclusive to the CTR‐ and Ma+ treatments, and seven genera (2.99%) were exclusive to the CTR+ and Ma‐ treatments. Phylum (Figure S3B) and family level profiles (Figure S4) showed high similarity in relative abundance among the analyzed groups, with Bacteroidota and Bacillota (both predominantly chemoheterotrophic phyla) being the most abundant. The mean relative abundance of Bacteroidota ranged from 43.18% (CTR+) to 47.49% (CTR‐), whereas Bacillota varied from 37.87% (Ma‐) to 38.56% (CTR‐). At the genus level, the relative abundance of the 50 most abundant genera (Figure S3C) did not reveal any specific sample grouping by treatment in the heatmap. The most abundant genera were *Phocaeicola* (14.21%), *Prevotella* (13.33%), and *Bacteroides* (6.17%), all of which belonged to the Bacteroidota phylum.

**Figure S3** Gut bacterial taxonomic composition and abundance in *Rhipicephalus microplus* shaped by early ecological interactions arising from exposure to *Metarhizium anisopliae*, assessed under conditions with and without *Anaplasma marginale* infection. (A) Venn diagrams illustrating the overlap of bacterial phyla and genera between the treatment groups (CTR‐, CTR+, Ma‐, and Ma+). (B) Bar graphs showing the relative abundance of bacterial phyla in the treatments. Low-abundance phyla were grouped under “Others.” (C) Heatmap displaying the distribution of the 50 most abundant bacterial genera, with abundance transformed into Z-scores. The overall abundance and corresponding phyla are indicated in the left column of the graph. Sample treatments are represented by color-coded bars above the graph. Hierarchical clustering of rows and columns was performed using Ward’s method.

**Figure S4** Relative abundance of bacterial families in Rhipicephalus microplus guts, comparing samples treated or untreated with *Metarhizium anisopliae* and infected or uninfected with *Anaplasma marginale*. Low-abundance families are grouped under “Others”.
